# The Genetic Basis of Moyamoya Disease

**DOI:** 10.1007/s12975-021-00940-2

**Published:** 2021-09-16

**Authors:** R. Mertens, M. Graupera, H. Gerhardt, A. Bersano, E. Tournier-Lasserve, M. A. Mensah, S. Mundlos, P. Vajkoczy

**Affiliations:** 1 Charité–Universitätsmedizin Berlin, corporate member of Freie Universität Berlin and Humboldt-Universität zu Berlin, Department of Neurosurgery, Berlin, Germany; 2 Vascular Biology and Signalling Group, ProCURE, Oncobell Program, Institut d’Investigació Biomèdica de Bellvitge (IDIBELL), L’Hospitalet de Llobregat, Catalonia Barcelona, Spain; 3 Integrative Vascular Biology Laboratory, Max-Delbrück Center for Molecular Medicine in the Helmholtz Association (MDC), Berlin, Germany; 4 Cerebrovascular Unit, Fondazione IRCCS Istituto Neurologico Carlo Besta, Milan, Italy; 5 Department of Genetics, NeuroDiderot, Lariboisière Hospital and INSERM UMR-1141, Paris-Diderot University, Paris, France; 6 Charité–Universitätsmedizin Berlin, corporate member of Freie Universität Berlin and Humboldt-Universität zu Berlin, Institute of Medical Genetics and Human Genetics, Berlin, Germany; 7 BIH Biomedical Innovation Academy, Digital Clinician Scientist Program, Berlin Institute of Health at Charité - Universitätsmedizin Berlin, Berlin, Germany; 8 Max Planck Institute for Molecular Genetics, RG Development & Disease, Berlin, Germany

**Keywords:** Moyamoya disease, Genetics, RNF 213, 17q25, Stroke

## Abstract

Moyamoya disease (MMD) is a rare cerebrovascular disease characterized by progressive spontaneous bilateral occlusion of the intracranial internal cerebral arteries (ICA) and their major branches with compensatory capillary collaterals resembling a “puff of smoke” (Japanese: Moyamoya) on cerebral angiography. These pathological alterations of the vessels are called Moyamoya arteriopathy or vasculopathy and a further distinction is made between primary and secondary MMD. Clinical presentation depends on age and population, with hemorrhage and ischemic infarcts in particular leading to severe neurological dysfunction or even death. Although the diagnostic suspicion can be posed by MRA or CTA, cerebral angiography is mandatory for diagnostic confirmation. Since no therapy to limit the stenotic lesions or the development of a collateral network is available, the only treatment established so far is surgical revascularization. The pathophysiology still remains unknown. Due to the early age of onset, familial cases and the variable incidence rate between different ethnic groups, the focus was put on genetic aspects early on. Several genetic risk loci as well as individual risk genes have been reported; however, few of them could be replicated in independent series. Linkage studies revealed linkage to the 17q25 locus. Multiple studies on the association of SNPs and MMD have been conducted, mainly focussing on the endothelium, smooth muscle cells, cytokines and growth factors. A variant of the RNF213 gene was shown to be strongly associated with MMD with a founder effect in the East Asian population. Although it is unknown how mutations in the RNF213 gene, encoding for a ubiquitously expressed 591 kDa cytosolic protein, lead to clinical features of MMD, RNF213 has been confirmed as a susceptibility gene in several studies with a gene dosage-dependent clinical phenotype, allowing preventive screening and possibly the  development of new therapeutic approaches. This review focuses on the genetic basis of primary MMD only.

## Definition

Moyamoya disease (MMD), first described in 1957 by Takeuchi and Shimizu [1] and characterized as a new disease entity in 1969 by Suzuki and Takaku [2], is a rare cerebrovascular disease characterized by progressive spontaneous bilateral occlusion of the intracranial internal carotid arteries (ICA) and their major branches (middle cerebral artery, MCA, and anterior cerebral artery, ACA) with compensatory capillary collaterals as an expression of pathologically increased angiogenic activity [3] resembling a “puff of smoke” (Japanese: Moyamoya) on cerebral angiography. Moyamoya collaterals may disappear with development of meningeal collaterals from the external cerebral arteries (ECA). Intracranial aneurysms are often found in patients with MMD. These pathological alterations of the vessels are called Moyamoya arteriopathy (MMA) or vasculopathy (MMV). A further distinction is made between primary and secondary MMD: primary or idiopathic MMD is defined as bilateral stenosis of ICA and their major branches that is neither of arteriosclerotic nor of inflammatory origin. The pathophysiology remains unknown. Similar vascular changes may also occur in the kidney, heart and/or other organs. Secondary MMD (also known as “moyamoya syndrome” or “quasi moyamoya disease”) is described as the typical angiographic findings associated with other autoimmune (e.g., Graves disease, systemic lupus erythematosus [4]), vascular (atherosclerosis), congenital (NF1 [5], Down syndrome [6], Turner syndrome [7]) and hematologic (e.g., sickle cell disease [8]) disorders. This review focuses on the genetic basis of primary MMD only.

## Epidemiology

The incidence of MMD has major ethnic differences. Initially, MMD was considered a specific disease in the Japanese population after its first description by Takeuchi and Shimizu [1]. MMD has now been observed in people of many ethnic backgrounds throughout the world, preferring the Asian race, primarily Japanese [9]. Baba et al*.* described a detection rate per year of 0.94/100.000 people and a prevalence of 10.5/100.000 people between 2002 and 2006 in Hokkaido, Japan. The ratio of female to male patients was 2.18. Two peaks for age of onset were described: between 5 and 9 years and between 45 and 49 years. Familial history was observed in 15.4% of patients. The detection rate and prevalence was higher than previously described, which has been interpreted as a result of better diagnostic possibilities, rather than an actual increase of the incidence [10]. The average detection rate per year and the prevalence in the Nanjing population (China) between 2000 and 2007 was described as 0.43/100.00 and 3.92/100.000 by Miao et al. [11]. In Korea, the incidence from 2007 to 2011 was 1.7 to 2.3/100.000, and the prevalence in 2011 was 16.1/100.000 [12]. MMD incidence in Japan, Korea, and China is higher than in Europe and North America [13]. In the USA, incidences per 100.000 patient years ranged from 0.05 [13, 14] in Iowa to 0.17 in Hawaii [13, 15]. The incidence rate of MMD among Asian Americans living in California is comparable to the incidence in Asia (0.28/100.000). Ethnic differences in MMD incidence appear to be maintained after immigration to the USA, with Asian Americans having the highest incidence [16]. Few population-based data are available from Europe, e.g., an incidence of 0.047 per 100.000 person years in Denmark [17]. The epidemiology in Western countries seems to differ from Asian countries and is characterized by a more pronounced female preponderance and a higher age at onset of disease [18].

## Clinical Presentation and Natural History

Symptoms are mainly attributed to changes in the cerebral blood flow resulting from stenosis of the ICA (ischemia) and from the fragility of the compensatory capillary collaterals (hemorrhage). The clinical manifestation seems to vary among geographic regions. In a Japanese cohort, the percentage of hemorrhagic manifestation was 21.0% with only one peak at 35–39 years, whereas the percentage of ischemic manifestation was 57.4% with two peaks: one at 5–9 years and the other at 45–49 years. Hemorrhagic manifestation is found in < 5% and 40% of cases amongst the pediatric and adult Japanese MMD population [10, 19]. In a Korean survey, the major clinical manifestations were ischemia in children (61.2% vs. 25.4% in adults) and hemorrhage in adults (62.4% vs. 9.1% in children). Among patients with ischemic symptoms, adults were more likely to have infarction than pediatric patients (80% vs. 39%) and pediatric patients were more likely to have transient ischemic attack (TIA) (61% vs. 25%) [20]. The phenotype in Western countries seems to differ from the phenotype in Asian countries and is characterised by a lower likelihood of hemorrhagic stroke as an adult [18]. In the USA, adults have much lower rates of hemorrhage as a presenting symptom (20%). The majority of affected adults and children in the USA presented with ischemic symptoms [6, 21]. Among European Caucasian MMD patients, 81% initially presented with ischemic and 8.5% with hemorrhagic manifestation. The rate of hemorrhagic manifestation amongst the pediatric group (≤ 18) was slightly higher than in the adult group (12% vs. 7.8%) [19]. According to the International Pediatric Stroke Study, MMD accounted for 8% of strokes in Caucasian children [22]. Another frequent symptom is headache, presumably due to dilatation of meningeal collateral vessels [23], as well as movement disorders [24]. Approximately 17.8% of Japanese patients are asymptomatic [10]. The natural history of untreated MMD is poor with a 73% rate of major deficit or death over two years following diagnosis in children [25]. The clinical symptomatology in MMD patients can be stratified by the Berlin grading system, which combines digital subtraction angiography (DSA), magnetic resonance imaging (MRI) and cerebrovascular reserve capacity (CVRC) as dependent factors associated with the occurrence of clinical symptoms [26].

## Neuroimaging

**Fig. 1:**
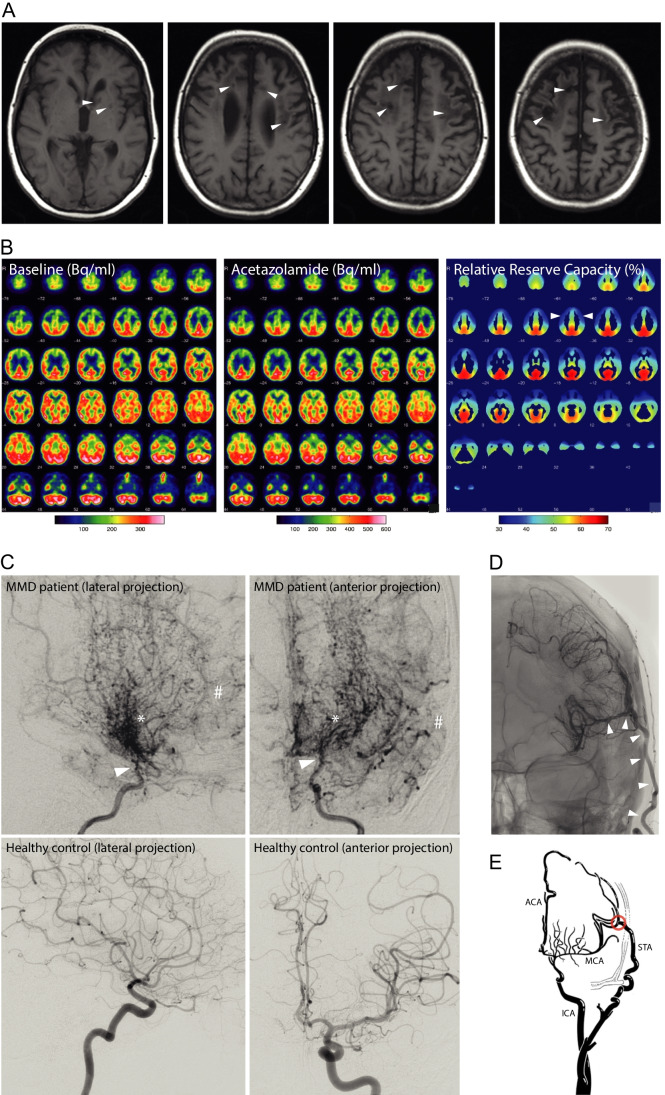
MRI, PET, and DSA findings in patients with MMD. A: MRI (T1) shows multi-stage infarction with extensive chronic infarcts frontally and in the left basal ganglia (▷) B: PET-MR brain examination with O-15-H_2_O (at baseline (Bq/ml), under acetazolamide challenge (Bq/ml) and calculated relative reserve capacity (%)) shows bilateral aggravation of the cerebrovascular reserve capacity in both ACA and MCA perfusion areas after acetazolamide challenge (▷). C: Supraaortal DSA (left ICA, upper row) shows supraophthalmic stenosis/occlusion of ICA (▷) with multiple hypertrophic Moyamoya collaterals (*) and restricted distal perfusion of ACA and MCA (#). In comparison, the lower row shows a DSA (left ICA) of a healthy patient. D: Postoperative angiography (anterior projection) of the left ECA after STA-MCA bypass surgery demonstrating bypass graft patency (▷). E: Illustration of STA-MCA bypass (red circle). Source figure E: 10.1161/STROKEAHA.117.018563; Courtesy of P. Vajkoczy. PET-MR was performed by the Department of Nuclear Medicine, Charité – Universitätsmedizin Berlin, Berlin, Germany. MRI and DSA was performed by the Department of Neuroradiology, Charité – Universitätsmedizin Berlin, Berlin, Germany

Diagnosis of MMD requires bilateral stenosis/occlusion of the ICA and its major branches as well as compensatory capillary collaterals, shown on cerebral angiography. Unenhanced computed tomography (CT) may show multiple and bilateral cortical and subcortical low density areas. Moyamoya vessels appear as flow voids on MRI. MRI often shows parenchymal ischemic lesions (Fig. 1A). Although CT-angiography or MR-angiography may pose the diagnostic suspicion, DSA is necessary to establish the diagnosis, identifying suitable vessels for bypass-surgery and to spot aneurysms (Fig. 1C). Cerebral blood flow (CBF) studies by positron emission tomography (PET) can identify areas of low perfusion with aggravation after acetazolamide challenge (causes vasodilatation, evaluates reserve capacity of cerebral blood flow and identifies areas at risk for future infarction, Fig. 1B).

## Treatment

No treatment can stop or reverse the primary disease progress. Recurring ischemic symptoms and impaired cerebral blood flow are the main indicators for treatment [27], but also determine the severity of symptoms and the risk of intervention [26, 28]. For asymptomatic patients, randomized trials are still missing to determine therapeutic procedures. Medical treatment with platelet inhibitors, anticoagulants, calcium channel blockers etc. has not proven to be of benefit, yet platelet inhibitors are broadly used to prevent ischemic insults.

Three different surgical revascularization methods are applied: direct revascularization by bypass surgery (e.g., STA-MCA-bypass, STA = superficial temporal artery; Fig. 1D + E), indirect and combined revascularization [29–32]. Currently, various surgical techniques are applied. Given the lack of randomised trials, there is neither clear evidence for the optimal timing of intervention nor which of these techniques is the most efficacious. However, current studies strongly indicate that surgical revascularization should be applied in patients with MMD of all age populations, if technically feasible [29].

## Pathological Hallmarks

As mentioned above, MMD is mainly characterized by progressive spontaneous bilateral occlusion of the terminal ICA and their major branches with compensatory capillary collaterals as an expression of pathologically increased angiogenic activity [3]. Histological examinations of the steno-occlusive arteries revealed fibrocellular thickening of the intima caused by proliferation of smooth muscle cells (SMC) with abnormal findings such as irregular undulation of the internal elastic lamina and significantly thinner media with absence of atheromatous plaques [33–35]. Moyamoya vessels are dilated perforating arteries, including preexisting and newly developed vessels as an expression of pathologically increased angiogenesis, vasculogenesis and arteriogenesis [36, 37]. In fact, MMD patients show more frequent angiogenesis events compared to patients with chronic cerebral ischemia of other etiologies such as atherosclerotic cerebrovascular disease (ACVD) [38, 39]. However, these newly formed vessels show impaired blood–brain barrier function, loss of endothelial cell integrity [40, 41] and various histopathological changes including fragmented elastic lamina, attenuated media, fibrin deposits in the wall and microaneurysms related to increased flow [6, 33, 42, 43]. Rupture of microaneurysms as well as increased fragility and leakage of the Moyamoya vessels are the main source of hemorrhagic stroke in MMD. Different dilatation patterns of the collaterals may be associated with an ethnic difference in the clinical presentation of MMD [44]. Thrombosis and collapse of the artery lumen can also be seen in the Moyamoya vessels, which could be the cause of ischemic symptoms [6, 33, 42]. Leptomeningeal vessels show attenuation or disruption of the internal elastic lamina and fibrous intimal thickening. These vessels seem not to be newly formed but are merely dilated preexisting vessels [45].

Given these pathological hallmarks, mutations of heterogeneous groups of genes could possibly be involved in the development of MMD. We speculate that mutations may lie in genes which

1. Affect smooth muscle cells (SMC) and
2. Affect the extracellular matrix (ECM), both leading to fibrocellular thickening of the intima and attenuation of the media
3. Lead to increased vascular plasticity and induce angiogenesis, vasculogenesis and arteriogenesis
4. Cause vessel fragility and vessel leakage and possibly weaken intra-extracellular contacts (could also be a result of point 3)
5. Are affected differently in various ethnic groups, explaining ethnic differences in vascular changes and disease patterns, such as frequency of intracerebral hemorrhage (point 4), different vessel occlusion patterns (point 1 + 2) and different collateral pathways (point 3).

## Genetics of MMD and Pathophysiological Features

**Fig. 2:**
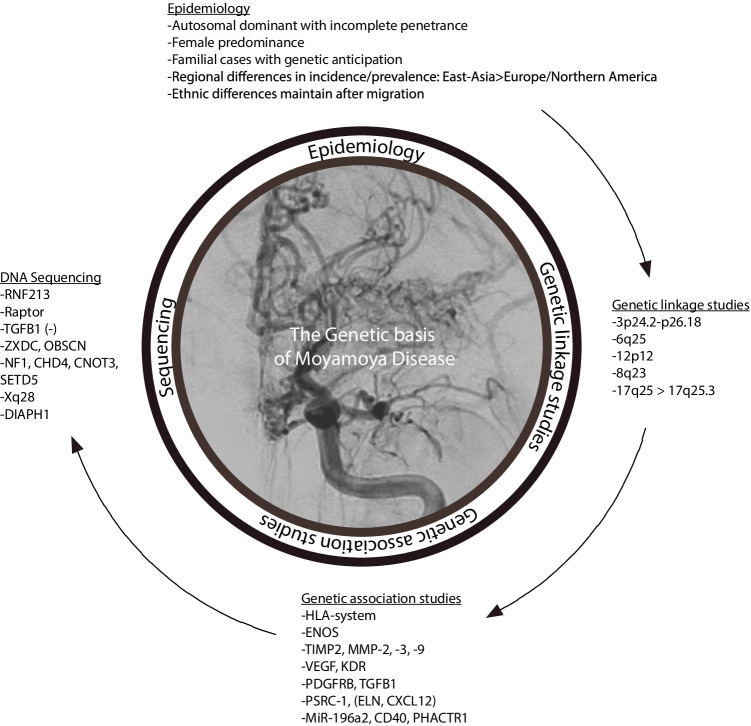
Overview of the genetic findings in MMD with a focus on different methodical approaches

The pathophysiology of MMD still remains unknown. MMD seems most likely to be a multifactorial disease, to which different factors such as environmental influences and genetic aspects contribute with varying degrees of impact. Different environmental factors such as radiation [46], bacterial infection [47], varicella zoster virus (VZV) [48], Epstein-Barr virus (EBV) and cytomegalovirus (CMV) [49] infections have been described to play a role in the pathophysiology of MMD. However, they could not be detected in the majority of affected patients. The incidence of MMD in patients of Asian descent living in non-Asian countries is comparable to the incidence in Asia [16], rendering a geographical or environmental influence unlikely. The concentration of affected patients in East Asian countries as well as familial cases strengthen the hypothesis that genetic factors have a major role in the development of the disease. Genetic variants found more frequent in some ethnic groups than others may explain the variable prevalence of MMD. Recently, genetic approaches have increasingly been used to clarify the pathogenesis of the disease—with success. In the following, we present the genetic aspects of the pathophysiology in thematic subgroups. Figure 2 summarizes the genetic findings in MMD focusing on the different methodical approaches that have been used.

## The Analysis of Epidemiological Data, Inheritance Patterns, and Data from Family Cases

The analysis of epidemiological data, inheritance patterns and data from family cases already suggests that genetic factors play a major role in the development of MMD. As described above, detection rate and prevalence rate vary among different ethnic populations, being the highest in East Asian countries. Family cases account for up to 15.4% of cases in Japan with major changes in epidemiological data within familial groups: the female preponderance was significantly more prominent in the familial than in the sporadic group with a male/female ratio of 1:5 vs. 1:1.6. Mean age at onset was significantly lower in familial (11.8 ± 11.7) than in sporadic cases (30.0 ± 20.9). In 8 parent–offspring pairs, mean age at onset was significantly lower in the second (7.2 ± 2.7) than in the first generation (30.7 ± 7.5) [10]. It was, hence, even suggested that familial MMD-cases could be associated with genetic anticipation, which would confirm a genetic pathophysiology [50]. To further determine the pattern of inheritance, Mineharu et al. examined 15 highly aggregated Japanese families (52 patients; 38 women and 14 men): all types of transmission between generations were observed and the mode of inheritance of familial MMD was reported to be autosomal dominant with incomplete penetrance with an increased rate of maternal transmission (3.44:1) [51]. An analysis of twin pairs suffering MMD revealed monozygotic predominance (4.7–7.5:1) with 5 of the 12 analyzed monozygotic twins showing discordant phenotypes [51]. This observation supports the concept of environmental factors triggering MMD in susceptible individuals. Recently, a population-based aggregation study in Korea, conducted to quantify the familial risk of MMD, was published by Ahn et al. [52]: by using a national data base, a cohort of 21.940.795 study subjects constituting 12 million families has been observed for a familial occurrence of MMD from 2002 to 2017. Incidence risk ratios (IRR) were calculated as the MMD incidence in individuals with affected first-degree relatives divided by the incidence in those without affected first-degree relatives. Summarized, the familial risk / IRR for MMD was 132 × higher in individuals with than in those without affected first-degree relatives, with 3.2% of the former and 0.02% of the latter developing MMD. The familial risk showed age dependence with a high peak in the younger age group, which then reduced with increasing age. Familial risk (IRR; incidence/10^4^person-years) markedly increased with the degree of genetic relatedness, being highest in individuals with an affected twin (1254.1; 230.0), followed by a sibling (212.4; 35.6), then mother (87.7; 14.4) and father (62.5; 10.4), indicating that genetic predisposition is the prevailing trigger in MMD pathogenesis [52]. Data on family cases in Caucasians are very rare and limited to a few case reports [53, 54]. The later onset and the lower familial occurrence demonstrated in Western patients may be accounted for the higher influence of genetic factors in Asian populations [18].

## Genetic Linkage Studies

Genetic linkage is the tendency of genetic loci that are positioned close together to be inherited together. In *linkage analyses*, the segregation of traits and diseases in families can be compared with the segregation of alleles of certain markers in meiosis. Genetic linkage analyses use the phenotypic findings of pedigree analyses and investigate their linkage to a genetic locus. If these loci appear to be linked to affected patients, it is assumed that disease-causing genes are located nearby. The statistical measure for linkage certainty is the logarithm of the odds score (LOD score). A linkage is considered significant if the LOD score is above 3. A LOD score of -2 or less indicates no linkage. Once the chromosomal subregion for a disease locus has been determined with a linkage analysis, databases can be searched for candidate genes in this region [55, 56]. Several linkage analyses regarding MMD have reported different linked regions. Linkage analyses under the assumption of autosomal recessive inheritance revealed that the D3S2387 and D3S3050 loci on chromosome 3 showed parametric LOD scores > 1.0. Nonparametric linkage analyses showed a nonparametric LOD score > 1.96 at loci between D3S2387 and D3S3050. These two loci are both located on chromosome 3p24.2-p26, a region that contains genes responsible for von Hippel-Lindau syndrome and Marfan syndrome [57]. Inoue et al. reported linkage of the marker D6S441 (6q25) to MMD [58]. Sakurai et al. reported significant evidence for linkage to 8q23 (D8S546) with a maximum LOD score of 3.6 and suggestive evidence for linkage to 12p12 (D12S1690) with a maximum LOD score of 2.3 by performing a genome-wide linkage analysis [59]. Yamauchi et al. described a new location of candidate genes causing familial MMD on chromosome 17q25. An initial screening of 4 Japanese families with 10 microsatellite markers showed a positive maximum LOD score of 0.32 at the D17S784 marker on chromosome 17q25. Then, detailed analyses of 24 families with 9 additional markers around D17S784 yielded a cumulative maximum LOD score of 3.11 for the marker D17S939 using a dominant transmission model. Multipoint linkage analysis revealed that the gene causing MMD was most likely to lie in the 9-cM interval between D17S785 and D17S836, with a maximum LOD score of 4.58 [60]. Shortly after this linkage has been described, Nanba et al. selected and sequenced 9 of 65 genes identified in the 9-cM region of D17S785-D17S836 in a pedigree of familial MMD, whose DNA samples showed a linkage to this region. No mutation was found in these genes [61]. Nevertheless, chromosome 17q25 remained a target region since only few genes in this region have been analyzed. Mineharu et al. suggested a major gene locus for dominant MMD on chromosome 17q25.3 by genome-wide linkage analysis. Significant linkage was observed on chromosome 17q25.3 (D17S704), with maximum LOD scores of 6.57 (narrow classification including definite MMD according to the RCMJ criteria) and 8.07 (broad classification, including any steno-occlusive lesions around the terminal portions of the ICAs, either unilaterally or bilaterally, even without findings of MMD vessels). The critical MMD gene location was defined within a 3.5-Mb region from D17S1806 to the end of chromosome 17q, encompassing 94 annotated genes. A mutational analysis of 4 candidate genes in this region was performed (BAIAP2, TIMP2, RAC3 and RAB40B), showing no significant mutations related to MMD. The locus reported by Yamauchi et al. [60] seemed not to be identical to the newly reported region. Moreover, the previously described loci 3p24-p26.18 and 8q23 [57, 59] satisfied the linkage exclusion criteria (LOD score <  − 2.0) [62]. Liu et al*.* further increased the LOD score of 8.07 for 17q25.3 to 9.67 and the linkage signal was narrowed to a 2.1 Mb region [63]. Linkage to the 17q25.3 locus has been further confirmed in several studies. Kamada et al. performed a linkage analysis focused on previously reported candidate loci (3p, 6q, 8q, 12p and 17q25). No locus with significant linkage with LOD > 3 could be confirmed. A suggestive linkage for the microsatellite maker D17S784 on chromosome 17q25 with a LOD score of 2.4 was observed [64]. Liu et al. confirmed linkage to 17q25.3 by genome-wide linkage analysis using 382 markers in 8 MMD families. Fine mapping revealed a 1.5-Mb locus bounded by D17S1806 and rs2280147, harboring 21 genes [65]. In summary, a number of linkage analyses have been conducted using either parametric or non-parametric methods. Five loci have been linked to MMD: 3p24.2-p26, 6q25, 8q23, 12p12, and 17q25. Except for the 17q25 locus, none of these loci has been confirmed, leading to the assumption that this locus may contain genes responsible for the development of MMD.

## Genetic Association Studies and Sequencing

Until the year 2000, the primary method of investigation in the discovery of genes for human diseases was through inheritance studies of genetic linkage in families. Highly useful towards single gene disorders, the results of genetic linkage studies in complex diseases proved hard to reproduce [66]. A suggested alternative to linkage studies was the genetic association study [67]*.* Association studies are observational case–control studies of genetic variants and markers in sporadic and/or familial cases to identify association with a trait, investigating whether an allele of a gene is found more often in individuals with the phenotype or disease of interest. Typically, association studies focus on association between single-nucleotide polymorphisms (SNPs) and diseases. Genome-wide association studies (GWAS) investigate the entire genome instead of pre-specified genetic regions [68]. With its introduction in the mid-1970s, standardized DNA sequencing revolutionized the decoding of the human genome. Classic methods such as Sanger sequencing and the Maxim-Gilbert method were further developed into a series of new techniques (next generation sequencing, NGS), which nowadays allow the nucleotide sequence in a DNA molecule to be decoded much faster and more cost-effective. Sequencing allows to determine whether a gene or the region that regulates a gene contains changes, called mutations or variants, that are linked to a disorder. Multiple studies on the association of polymorphisms to MMD have been conducted, mainly focussing on endothelium, smooth muscle cells, cytokines and growth factors. Sequencing revealed several new candidate genes, which further contributed to the pathophysiological knowledge about MMD.

### The Human Leukocyte Antigen (HLA) System

The human leukocyte antigen (HLA) system is a group of proteins that are encoded by a gene complex called the major histocompatibility complex (MHC, located on chromosome 6p21.3, encoding the class I HLA-A/B/C and class II HLA-DR/DQ/DP), responsible for the regulation of the immune system and identifying self versus non-self. HLA genes are highly polymorphic. In general, the inheritance of a disease-associated HLA allele is a risk factor that increases a patient’s likelihood of developing the disease [69]. HLA genes have been studied in Asian and European MMD patients and several associations have been described. One of the first HLA–MMD association studies conducted by Kitahara et al. revealed a significant association with HLA-AW24, -BW46 and – BW54 with a relative risk (RR) of 3.83, 6.50, and 3.58 in Japanese MMD patients and controls [70]. Aoyagi et al. performed HLA genotyping in Japanese MMD patients and controls, revealing a significant positive association between MMD and HLA-B51 with a RR of 3.7. The previously described HLA-AW24, -BW46 and -BW54 frequencies [70] did not differ between MMD patients and healthy controls [71]. Inoue et al. genotyped HLA class II gene alleles in unrelated Japanese MMD patients and controls, showing a significant positive association between MMD and HLA-DQB1*0502 and a significant negative association between MMD and DRB1*0405 and DQB1*0401 with RRs of 3.27, 0.35, and 0.40 [72]. To confirm these results, Han et al. genotyped HLA class I and II alleles in Korean MMD patients and controls. The frequency of the HLA-B35 allele was significantly increased in the MMD patients compared to the controls with a RR of 4.2. HLA-B35 was most significantly increased in female patients with late-onset MMD. The above mentioned previous findings on HLA-B51, DQB1*0502, DRB1*0405, and DQB1*0401 in the Japanese cohort could not be replicated in this Korean cohort [73]. To investigate whether there is a difference in HLA class II association between familial and non-familial MMD patients, HLA class II alleles were genotyped in Korean children with MMD including familial cases and controls. The frequencies of HLA-DRB1*1302 (70.0%) and DQB1*0609 (40.0%) were significantly increased in familial MMD patients compared to both controls (vs. 15.5%, odds ratio (OR) = 12.76; vs. 4.3%, OR = 14.67) and non-familial MMD patients (vs. 14.8%, OR = 13.42; vs. 1.9%, OR = 35.33). There was no significant difference in the frequencies of HLA-DRB1 and -DQB1 alleles in non-familial MMD patients vs. healthy controls [74]. To investigate HLA association in Caucasian patients, Kreamer et al. analyzed DNA of Caucasian patients (22 MMD + 11 MMA). HLA-A, -B, and -DRB1 frequencies were compared with those of 13.386 German blood donors, whereas the HLA-DQB1 frequencies were compared with US American National Marrow Donor Program (NMDP) data from 15.470 different Caucasian donors. Significant disease association was found for HLA-DRB1*03 (for all 33 MMD + MMA patients (RR 4.443) and for all 22 patients with MMD (RR 5.391)) and for HLA-DRB1*13 (for all 33 patients (RR 4.268) with a lower significant frequency in those patients with MMD (RR 4.277)). Moreover, HLA-A*02, HLA-B*08, and HLA-DQB1*03 showed lower significantly elevated frequency in the 33 patients compared with the controls but not in the subgroup analysis. Despite HLA-DRB1*13, HLA markers previously described in the Asian literature were not replicable with high levels of significance: HLA-DQB1*05, -B35 and –AW24 only showed weakly significantly elevated frequencies in the Caucasian cohort [75]. More recently, Tashiro et al. identified HLA-DRB1*04:10 as a new risk allele and HLA-DRB1*04:10 – HLA- DQB1*04:02 as a risk haplotype in Japanese MMD patients. This result was again inconsistent with previous results [76]. However, the causative role of HLA polymorphisms in the molecular immunopathogenesis of HLA associated diseases remains often poorly understood. Association may be due to HLA presentation of altered or disease-triggering self-antigens, cross reactivity between self and foreign antigens or autoimmune response to self-antigens due to erroneous T cell selection [77], but a causal relationship in MMD is not understood yet. Altogether, the reported results and the fact that most findings could not be reproduced in independent studies may suggest that the association between HLA antigens and MMD differ between ethnic groups and that the different HLA alleles may not be significant susceptibility factors of MMD rather than genetic markers.

### Smooth Muscle Cells (SMC) and Endothelial Nitric Oxide Synthase (eNOS)-Derived Nitric Oxide (NO)

The major function of vascular smooth muscle cells (SMC) is to contract in response to the stretch resulting from pulsatile blood flow. Endothelial nitric oxide synthase (eNOS)-derived nitric oxide (NO) is one of the principal molecules in vasoregulation. Endothelial NO is responsible for regulation of SMC and endothelium-dependent vasodilatation. Moreover, it is involved in the inhibition of leukocyte and platelet adhesion and attenuation of inflammatory mediators [78]. NO has protective as well as cytotoxic effects after cerebral ischemia [79]. Therefore, an understanding of eNOS polymorphisms may help to explain variations in the clinical aspects of MMD. Park et al. conducted a case–control study on eNOS polymorphisms, suggesting that pediatric and adult-onset MMD may have different genetic backgrounds. In this study, 93 Korean MMD patients and 328 healthy controls were divided into pediatric (< 20 years) and adult (≥ 20 years) groups and further according to MRI findings into ischemic and hemorrhagic groups. No differences in four eNOS polymorphisms (eNOS − 922A > G, − 786 T > C, variable number of tandem repeat (= VNTR) polymorphism 4a4b, and 894G > T) were observed between the combined MMD patient group and a control group. However, the 4a4b sequences were less frequent in the adult patient group and the ischemic group. Compared to the control group, the A-4b-G haplotype was seen more frequently in the adult patient group, suggesting that these genetic differences can affect age-specific clinical characteristics [80]. Following linkage analyses and confirming linkage to chromosome 17q25.3, Liu et al. performed sequencing of three genes encoded in this region, revealing one newly identified polymorphism, ss161110142 G/A, located at the transcription site of the Raptor gene. Raptor is associated with vascular SMC proliferation and intimal expansion. This polymorphism was genotyped in 34 families, revealing that every affected individual in these tested families was found to carry the ss161110142 A allele, which showed complete segregation. The authors conducted a case–control association study for MMD performed in different ethnicities. The ss161110142 A allele was found much more frequent in cases than in the controls: 26% vs. 1% in the Japanese (90 patients vs. 384 controls), 33% vs. 1% in the Koreans (41 patients vs. 223 controls) and 4% vs. 0% in the Chinese (23 patients vs. 100 controls). The allele was not found in the Caucasian group [63].

#### Matrix Metalloproteinases (MMPs) and Tissue Inhibitors of Metalloproteinases (TIMPs)

The balance between matrix metalloproteinases (MMPs) and tissue inhibitors of metalloproteinases (TIMPs) is critical for blood brain barrier (BBB) integrity [81], vascular injury and repair and by degrading the neurovascular matrix, MMPs promote BBB damage, oedema, and hemorrhage [82]. For example collagen IV, the essential substrate of MMP-9, is markedly affected in MMD vessel specimens compared to those obtained from atherosclerotic cerebrovascular disease patients [83]. Disruption of the balance between MMPs and TIMPs can result in aberrant ECM and SMC dynamics [84], which could facilitate the pathological hallmarks 1 and 2 of MMD. Kang et al. investigated SNPs of the promoter regions, exon–intron junctions and the exons of the TIMP2 and TIMP4 genes in 11 Korean familial MMD patients, 50 Korean non-familial MMD patients and 50 control patients. The genes encoding TIMP2 and TIMP4 span chromosomes 3p24.2-p26 and 17q25, which have been linked to familial MMD before [57, 60]. A significantly higher frequency of the heterozygous G/C genotype was found in the TIMP2 promoter region at position −418 in familial MMD (rs8179090). This genotype was observed in 9 of 11 patients with familial MMD, in 16 out of 50 non-familial MMD patients and in 14 out of 50 control patients. OR between familial and non-familial MMD was 9.56 (95% confidence interval, 1.85–49.48; *p* = 0.005) and 10.50 between familial MMD and control patients (95% confidence interval, 2.02–54.55; *p* = 0.001) with no significant differences between non-familial MMD patients and controls. The base at position –418 corresponds to the site that binds the transcription factor Sp1. Thus, this variation may modify TIMP2 transcription by modifying Sp1 binding [84]. Paez and Yamamoto analyzed the genotype frequency of the same SNP in 7 Japanese familial MMD patients, 41 non-familial MMD patients and 52 control patients. The results revealed only 1 familial MMD patient (14%) with the G/C heterozygous genotype; the other 6 demonstrated the G/G homozygous genotype. There was no significant difference in the SNP frequencies of familial and non-familial MMD patients and controls, thus the results of Kang et al. could not be replicated in Japanese MMD patients [85]. The promoter polymorphism −418G > C in the TIMP2 gene could also not be replicated in another study with Japanese MMD patients from Mineharu et al. [62]. Roder et al. did not find a different frequency of the SNP rs8179090 C/G in the TIMP2 gene in 40 European MMD patients and 68 controls [86]. Li et al. conducted an association study of 5 promoter SNPs in the MMP-2, -3, -9 and -13 genes and 1 promoter SNP in the TIMP2 gene in 208 Chinese Han MMD patients, including 31 familial MMD patients and 224 control patients. In a dominant genetic model, the frequency of the MMP-3 5A/6A and 5A/5A genotypes was significantly lower in MMD patients (OR = 0.57, 95% confidence interval 0.38–0.86, *pcorr* = 0.042) than in healthy controls. Subjects with the 5A/6A and 5A/5A genotypes had a 43% reduced risk of MMD compared to those with the homozygous 6A/6A genotype. The 6A allele frequency was higher in patients than in controls (85.3 vs. 78.8%; OR = 0.64, 95% convidence interval 0.45–0.91, *p* = 0.013), but this was not significant after Bonferroni correction (*pcorr *= 0.078). Significant differences of the MMP-3 5A/6A polymorphism (rs3025058) were also detected between familial MMD patients and controls both in the dominant genetic model (OR = 0.23, 95% confidence interval 0.08–0.68, *pcorr* = 0.048) and the additive genetic model (OR = 0.24, 95% confidence interval 0.08–0.69, *pcorr* = 0.048). No difference in the MMP-3 5A/6A polymorphism distribution was found among patients with different clinical and age characteristics. The allele and genotype distributions of the other 5 SNPs, including TIMP2 rs8179090, did not reveal any association. Thus, this polymorphism in the MMP-3 promoter might be associated with susceptibility to familial and non-familial MMD in the Chinese Han population [87]. Wang et al. performed an association study of polymorphisms of PDGFRB, MMP-3, TIMP2, and RNF213 genes in 96 non-familial Chinese Han MMD patients and 96 controls. The loci rs3025058 in MMP-3 and rs8179090 in TIMP2 were analyzed, but no significant association between the polymorphisms and non-familial MMD was observed [88], whereas the association of rs8179090 in TIMP2 initially described by Kang et al*.* was found in Korean MMD patients [84]. Park et al. performed an association study focused on 6 SNPs in MMPs-2, -3, and -9 and TIMP2 in 107 Korean MMD patients (no familial cases were included) and 243 controls. A significantly higher frequency of TIMP2 -418 G > C (rs8179090) was found in MMD patients, corroborating previous results reported by Kang et al. [84]. The dominant (GG vs. GC + CC) genotype of TIMP2–418 was more frequent in patients with MMD. The MMP-9 Q279R (rs17576) GA + AA genotype showed a protective effect for MMD. The GA/CC MMP-2 -1575(rs243866)/-1306 (rs243865) genotype was significantly more prevalent in MMD patients [89]. Ma et al. performed a case–control study to examine association between MMP-3 polymorphisms and the risk of MMD, comparing 86 Chinese Han MMD patients and 86 controls. MMP-3 6A/6A genotype (OR = 1.93, 95% confidence interval 1.00–3.72; *p* = 0.05) and 6A allele frequencies (OR = 1.78, 95% confidence interval 1.00–3.14; *p* = 0.05) in the MMD group were significantly higher than those in the control group. Furthermore, a meta-analysis was performed including two former studies that evaluated the association between MMP-3 polymorphism and the risk of MMD [87, 88], analysing a total of 796 Chinese Han MMD patients. This meta-analysis revealed that one copy of the 6A allele significantly increases the risk of MMD (OR = 1.64, 95% confidence interval 1.26–2.13, *p* = 0.0002). Moreover, the risk of MMD was higher in the 6A/6A genotype than in the genotypes of 6A/5A and 5A/5A (OR = 1.79, 95% confidence interval 1.32–2.42, *p* = 0.0002) [90]. Recently, Wang et al. published a meta-analysis, evaluating seven polymorphisms in 4711 MMD patients and 8704 controls in 24 studies. This meta-analysis showed an inverse association of TIMP2 rs8179090, MMP-2 rs243865 and MMP-3 rs3025058 with MMD [91]. Summarized, the presented studies reported associations between MMD and polymorphisms located in the genes or promoters of TIMP2, MMP-2, -3 and -9. Nevertheless, it is difficult to draw conclusions about the significance of these results and their influence in the pathophysiology of MMD due to conflicting results of the published studies and lack of reproductions.

#### Cytokines and Growth Factors: Vascular Endothelial Growth Factor (VEGF)

Several cytokines and growth factors have been associated with MMD. Vascular endothelial growth factor (VEGF) expression has been reported to be significantly increased in MMD patient’s dural tissue [92] and plasma VEGF concentrations in MMD patients were significantly higher than those in healthy controls [93, 94]. VEGF is crucial in endothelial cell proliferation, migration, survival and vascular permeability during vasculogenesis and angiogenesis [95]. VEGF binds to tyrosine kinase cell receptors: VEGFR-1 (Fms-like tyrosine kinase = Flt-1), VEGFR-2 (kinase insert domain receptor = KDR) and VEGFR-3 (Fms-like tyrosine kinase 4 = Flt-4), with VEGFR-2 having the strongest pro-angiogenic activity [96]. Changes in the corresponding gene could lead to the pathological hallmark 3. Park et al. conducted a case–control study to investigate whether VEGF (-2578C > A, -1154G > A, -634G > C, and 936C > T) and KDR (-604 T > C, 1192G > A, and 1719 T > A) polymorphisms are associated with MMD in 107 Korean patients and 243 healthy control subjects. No differences were observed in VEGF or KDR polymorphisms between the MMD patients and the control subjects. The subjects were divided into pediatric (< 18 years) and adult (≥ 18 years) groups, revealing that the VEGF-634CC genotype occurred significantly less frequently in the pediatric MMD group. The frequencies of the KDR polymorphisms in both the pediatric and adult subgroups were not significantly different, whereas the KDR -604C/1192A/1719 T haplotype significantly increased the risk of pediatric MMD. Among the 64 surgical patients (indirect bypass surgery), collateral vessel formation was evaluated after two years. Patients with the CC genotype of VEGF -634 had better collateral vessel formation after surgery, altogether suggesting that the VEGF -634G allele is associated with pediatric MMD and poor collateral vessel formation [97]. Soluble VEGF receptor (sVEGFR) -1 and -2 may play a role in the pathogenesis of MMD as they seem to influence postoperative collateralization. Patients with good collateral formation appeared to have lower sVEGFR-1 and sVEGFR-2 levels prior to and at day seven following bypass surgery when compared with patients with worse collateralization [98].

#### Cytokines and Growth Factors: Platelet-Derived Growth Factor (PDGF), PDGF Receptor Beta (PDGFRβ), and Transforming Growth Factor beta (TGFβ)

The platelet-derived growth factor (PDGF, e.g., PDGF-B/-BB) and the PDGF receptor beta (PDGFRβ, encoded by PDGFRB), as well as transforming growth factor beta (TGFβ, encoded by TGFB) signalling regulate endothelial and mural cells and the interactions of pericytes/SMC with endothelial cell tubes and thereby blood vessel differentiation, formation and tightness [99, 100]. Alterations of the corresponding genes could lead to the pathological hallmarks 3 and 4. PDGF has been shown to have an angiogenic effect and has also been implicated in the regulation of the tonus of blood vessels [101]. PDGF-B induces mural cell fate in undifferentiated mesenchymal cells and stimulates proliferation of vascular SMC. PDGF-B is secreted as a homodimer from the endothelium of angiogenic sprouts, serving as an attractant for comigrating pericytes, which express PDGFRβ4 [99]. In vitro studies reported a decrease in growth of cultured SMC derived from arteries of MMD patients as response to PDGF-BB, a fact explained by the reduced number of the PDGF receptors on these SMC [102, 103]. MMD patients exhibited significantly higher plasma concentrations PDGF-BB [94], which may be explained as a compensative mechanism to a systemically decreased amount of PDGFB receptors [104]. TGFB1 is a multifunctional protein associated with regulation of cell growth and differentiation and a potent angiogenic factor as well as a major modulator of the expression of connective tissue genes [105]. Higher concentrations of TGFB1 were found in blood serum and SMC of MMD patients [105], pointing to a possible role in the pathophysiology of MMD. In 2010 and 2011, Roder et al. published the first genetic studies on Caucasian MMD patients from central Europe (mainly Swiss and German). Their first study was based on the examination of 13 SNPs in or upstream of 4 genes of cytokines and growth factors with known abnormalities in MMD patients (BFGF, CRABP1, PDGFRB and TGFB1), comparing 40 DNA samples of MMD patients to 68 healthy controls. They found a significant association of two SNPs: rs382861 [A/C] (*p* = 0.0373, OR = 1.81, 95% confidence interval  1.03–3.17) in the promoter region of PDGFRB and rs1800471 [C/G] (*p* = 0.0345, OR = 7.65, 95% confidence intercal  0.97–59.95), located in the first exon of TGFB1. Rs1800470, also located in exon1 of TGFB1, showed tendencies towards a risk allele [104]. The second study was based on the description of common histopathological changes in the vessels of MMD patients and patients with atherosclerotic disease. Seventeen SNPs in or adjacent to 11 genes that had previously been described to be associated with atherosclerotic disease (ELN, LIMK1, CDKN2A/B, CXCL12, Pseudogene ENSG00000197218, PSRC-1, MTHFD1L, SMAD3, MIA3, PDGF-B, TIMP2) were analyzed, again comparing 40 DNA samples of European MMD patients to 68 healthy controls. Strong association of the SNP rs599839 [A/G] (OR = 2.17, 95% confidence interval 1.17-4.05; *p* = 0.01) with the risk allele G located on chromosome 1p13.3 in the 3' UTR region of the PSRC-1 gene and a trend towards significance for three other SNPs (rs8326, rs34208922, rs501120) in or adjacent to the genes ELN and CXCL12 were found [86]. PDGFRB and TGFB1 have been further analyzed in different association studies including further ethnicities. Liu et al. performed genotyping of the previously described SNPs rs1800470 and rs1800471 in 45 Japanese MMD patients and 79 healthy controls. The association of rs1800471 and tendency toward significance of rs1800470 previously described in the European cohort [104] could not be replicated in the Japanese cohort. Furthermore, the complete first exon of TGFB1 was genotyped in 40 European MMD patients and 68 healthy controls, whereby no new disease-associated nor other genetic variations could be identified. This in turn makes a role of this TGFB1 exon in the pathogenesis of MMD unlikely [106]. In the association study of polymorphisms of PDGFRB, MMP-3, TIMP-2 and RNF213 genes in 96 non-familial Chinese Han MMD patients and 96 controls performed by Wang et al., the SNP rs3828610 in PDGFRB was analyzed, showing no significant association to non-familial MMD [88].

#### Several other SNPs and genes

Several other SNPs and genes have been associated with MMD, for example the frequency of the CT + CC genotype in the SNP rs11614913 in miR-196a2 was significantly higher in Korean MMD patients, which suggests that miR-196a2 may play a role in the pathogenesis of MMD [107]. MMD has been associated with autoimmune disease, e.g., Graves disease and systemic lupus erythematosus [4] and Kawasaki disease [108]. Shen et al. analyzed patients’ DNA of 48 Chinese pediatric MMD patients and 50 healthy controls for five SNPs in B lymphoid tyrosine kinase, CD40 and coatomer protein complex beta-2 subunit, which had been associated with Kawasaki disease before. Two SNPs of CD40 were associated with MMD: the polymorphisms rs4813003 major allele CC and rs1535045 minor allele TT were significantly associated with MMD, providing evidence for autoimmune dysfunction in MMD patients [109]. Yang et al. reported that the SNP (c.13185159G > T, p.V265L) on PHACTR1 was highly associated with the disease progression of MMD in Chinese MMD patients. Silencing of PHACTR1 reduced survival of human coronary artery endothelial cells [110]. The most highly enriched variant in 10 of 68 Caucasian MMD patients (14.7%) in a study by Shoemaker et al. was rs16837497 (ZXDC, p.P562L, involved in MHC Class II activation). This variant has a frequency of 4% in the Caucasian population. Furthermore, collapsing variant methodology ranked OBSCN (involved in myofibrillogenesis) as most enriched in Caucasian and non-RNF213 founder mutation cases, revealing alternative candidate genes and thus confirming the multiethnic genetic landscape of MMD [111]. To further identify genes involved in the pathogenesis of MMD, exome sequencing was pursued in 197 unrelated Caucasian patients (172 bilateral, 24 unilateral, and 1 unknown) and 125 relatives (21 affected and 104 clinically healthy), including a total of 39 trios. 39 genes harbouring rare de novo variants were identified in 26 MMA trios (2 trios had 3 de novo variants, 12 trios had 2 de novo variants, 12 trios had 1 de novo variant). Three of these variants were in RNF213 and one was a variant of unknown significance in NF1. In four MMA patients, de novo variants in three genes, CHD4, CNOT3 and SETD5, were identified, previously found to harbour pathogenic de novo variants in children with developmental disorders [112]. Peng et al. investigated gene dysregulation in peripheral blood of twelve Chinese MMD patients and compared it with other vascular disorders. A total of 533 differentially expressed genes (DEGs) were identified for MMD. Up-regulated genes were mainly involved in ECM organization, whereas downregulated genes were primarily associated with inflammatory and immune responses [113]. Rare de novo copy number variation (CNV) screening was performed by Aloui et al. in 13 MMD trios using whole exome sequencing (WES) read depth data and whole genome high density SNP array data. WES and SNP array data from an additional cohort of 115 unrelated MMD patients were used to search for recurrence of CNVs. Two de novo CNVs were identified in two unrelated probands. One of these CNVs, located on Xq28, was detected in two additional families. The critical region contains five genes, including MAMLD1, a major NOTCH coactivator [114]. Recently, DIAPH1 (mammalian diaphanous-1) has been described as a novel MMD risk gene in non-East Asian individuals with sporadic MMD by impairing vascular cell actin remodelling [115].

## RNF213

### First Description as Susceptibility Gene for MMD

In 2011, the first GWAS on MMD revealed an association with a new primary susceptibility gene for MMD, *RNF213,* also known as *mysterin*, encoding the 591-kDa protein “ring finger protein 213” with two AAA + modules (ATPases associated with diverse cellular activities) and a single RING finger ubiquitin ligase domain [116]*.* A GWAS of 785.720 SNPs was performed, comparing 72 Japanese MMD patients including 8 familial MMD patients with 45 control subjects. A strong association of chromosome 17q25-ter with MMD risk was shown and further confirmed by a locus-specific association study using 384 SNPs in the 17q25-ter region. Mutational analysis of RNF213 (located on chromosome 17q25) revealed a founder variant, p.R4859K (single base substitution c.14576G > A in exon 60 of RNF213) in 95% of MMD families, 73% of non-familial MMD cases and 1.4% of controls. The carrier frequency of p.R4859K in Japan was assumed to be 1/72. This variant significantly increased the risk of MMD (OR 190.8). The p.R4859K variant was not present among Caucasian patients or controls [64] nor in Korean patients [117]. In the same year, Liu et al. also provided evidence on the involvement of RNF213 in genetic susceptibility to MMD. Genome-wide linkage analysis in 8 three-generation Japanese families with MMD confirmed significant linkage to 17q25.3. Exome analysis revealed the missense variant p.R4810K (single base substitution c.14429G > A, rs112735431) in RNF213 in the 1.5-Mb locus of 8 index cases in these families. Sequencing RNF213 in 42 further patients confirmed p.R4810K and revealed it to be the only unregistered variant. Genotyping 39 SNPs around RNF213 revealed a founder haplotype transmitted in 42 families. A further conducted case–control study demonstrated strong association of p.R4810K with MMD in East Asian populations (251 cases and 707 controls) with an OR of 111.8. The population attributable riRNF213 Variants in Caucasianssks and OR in the Japanese (145/161 = 90%; 339.94) and Korean (30/38 = 79%; 135.63) populations were higher than those in the Chinese population (12/52 = 23%; 14.70). The p.R4810K variant was not present among Caucasian patients or controls [65]. It is important to note that there are several transcripts of the RNF213 gene: NM_001256071.3:c.14429G > A; p.R4810K and XM_005257545.1:c.14576G > A; p.R4859K both correspond to the SNP rs112735431. It is therefore the same variant that has been described in these two initial publications.

### Distribution of the Founder Variant p.R4810K in East Asia

Association of p.R4810K was further confirmed in Japanese, Chinese and Korean patients in large-scale case–control studies [117–124]. The carrier rates of p.R4810K in MMD patients were greatly discrepant in different populations. In Japan and Korea, the distribution of the p.R4810K variant was similar with a frequency of 67.4–90% and a frequency of homozygous carriers of 5.3–7.9% [65, 117, 118, 125, 126]. The distribution of the p.R4810K variant in China is quite different from Japan and Korea with much lower rates of homozygous and heterozygous carriers than wild type carriers. A large-scale case–control study in Chinese Han patients reported p.R4810K variant in 22/170 patients (13%) including 21 heterozygotes and a single familial homozygote [127]. The lower carrier rates in Chinese patients were further confirmed by Wang et al. with frequencies of 0.5% for homozygous, 22.4% for heterozygous and 76.1% for wild type carriers [122]. Liao et al. conducted a meta-analysis, again confirming association of RNF213 p.R4810K, which significantly increased familial MMD risk in the Japanese, Korean and Chinese population (dominant model ORs 1802.44, 512.42, and 1109.02), with 5–36 times larger effect sizes than that for sporadic cases (dominant model ORs 134.35, 99.82, and 30.52). The effect sizes of p.R4810K to sporadic MMD were 3–4 times larger in Japan and Korea than those in China [128]. The RNF213 p.R4810K variant segregates with MMD in Asian families [65]. In East Asian MMD patients negative for p.R4810K, other rare RNF213 variants have been described and conferred susceptibility to MMD (see below). Approximately 20% of Japanese MMD patients do not harbour susceptibility variants of RNF213, indicating the presence of other susceptibility genes for MMD [119].

### RNF213 Variants in Caucasians

Neither the p.R4810K nor the p.R4859K variant have been detected in Caucasian patients, suggesting that these variants are Asian-specific RNF213 founder variants and may contribute to the high incidence of MMD in East Asia [129]. No predominant susceptibility variants have been identified in Caucasian MMD patients so far [130]. Several rare RNF213 variants distinct from p.R4810K and p.R4859K have been reported in Caucasian MMD patients with no further evidence of association in large patient cohorts [54, 65, 129, 131–134], such as p.D4013N, p.R4019C, p.E4042K, p.V4146A, and p.W4677L in Slovakian and Czech MMD patients [135]. Guey et al. investigated whether rare RNF213 variants are associated with increased risk of MMD and provided significant evidence for a positive association of rare RNF213 missense variants and MMD in European patients with an even stronger association in early onset and/or familial cases. Interestingly, these rare variants found in the Caucasian MMD patients are significantly grouped in a C-terminal region of RNF213 that includes the RING finger domain [136]. The named studies confirmed RNF213 as a causative gene for MMD not only in Asian but also in Caucasian patients.

### Penetrance and Pathophysiological Relevance

A meta-analysis of five previously published studies [64, 65, 118, 127, 137], including 421 (Chinese/East Asian/Caucasian) patients and 1214 controls for the p.R4810K polymorphism and 398 Japanese patients and 765 controls for the p.R4859K polymorphism confirmed strong associations between the p.R4810K polymorphism of the RNF213 gene and MMD (pooled OR 157.53 vs. 92.03) [138]. A further meta-analysis confirmed association of rs112735431 (= variant p.R4810K) polymorphism with MMD [139]. In conclusion, the R4810K/R4859K variant is found in East Asian patients and distinct other variants have been observed in Asians and Caucasians [54, 64, 65, 88, 111, 119, 120, 127–129, 131–136, 140]. GnomAD V2 (the Genome Aggregation Database of healthy individuals, aggregating 15.708 whole genomes and 125.748 exomes) shows a global allele frequency of 0.0002 for the rs112735431 allele (= variant p.R4810K); this allele frequency reaches a maximum of 0.01 in Korean individuals. In above mentioned studies, p.R4810K was observed in 1–2% of the general East Asian population [65, 141]: p.R4810K was detected at allele frequencies of 0.43%, 1.36%, and 1.36% in the Chinese (with heterogeneous distribution), Japanese and Korean populations, respectively. Frequencies of 1.00% in 4 of 11 investigated locations in China and frequencies of 1.00–1.72% in all investigated locations in Japan and Korea were reported. The estimated total numbers of carriers were 11.41 million for the Chinese, 3.39 million for the Japanese and 1.36 million for the Korean population. The number of patients with MMD, which was estimated at approximately 1 per 300 carriers of p.R4810K, was considered to be 53.800 in East Asian populations (38.000 in the Chinese, 4.500 in the Korean and 11.300 in the Japanese population) [141]. It is now very clear that this variant is associated with and substantially increases the susceptibility to MMD, but the penetrance of this variant is low as numerous carriers are unaffected and is estimated to be close to 1/150–300 (based on the respective frequencies of this allele in MMD patients versus the healthy Japanese population). Thus, other triggers are needed to develop the disease, such as environmental factors. Therefore, the reported RNF213 variants should be considered as susceptibility variants rather than a causing variants for MMD. RNF213 is a large gene (encoding 5207 amino acids) and harbours a number of variants in healthy subjects as well as in patients and false attribution of pathogenicity can have severe consequences for patients, resulting in incorrect prognostic, therapeutic or reproductive advice [142, 143].

### Genotype–Phenotype Correlation

After describing RNF213 as a new susceptibility gene, further studies have addressed the question whether different doses of RNF213 variants differ in the clinical phenotype of MMD. Besides the mentioned differences in the distribution of the variants, there is also a difference in the clinical phenotype between East Asian countries. In Japan and Korea, p.R4810K homozygous carriers showed a similar and more severe phenotype. Compared with heterozygotes in a Japanese MMD cohort, c.14576G > A (= p.R4859K) homozygotes had a significantly earlier age at onset. 60% of homozygotes were diagnosed before the age of four and all had infarctions as the first symptom. Infarctions at initial presentation and involvement of posterior cerebral arteries (PCA) were significantly more frequent in homozygotes than in heterozygotes and wild types, both known as poor prognostic factors in MMD. The incidence rate for homozygotes of the c.14576G > A variant was calculated to be > 78%. Thus the homozygous c.14576G > A variant was suggested to be a possible genetic biomarker for predicting a severe form of MMD [118]. These findings were further confirmed in Japanese MMD siblings. The c.14576G > A homozygous brother showed an early onset age and rapid disease progression with significant neurological deficits and a severe distribution of vasculopathy, whereas the heterozygous sister showed a late onset age and mild clinical course with a limited distribution of vasculopathy. Therefore, different doses of this RNF213 variant seem to influence the clinical phenotype, even in individuals with a similar genetic background [144]. Another study involving 165 Korean MMD patients confirmed dosage dependence of clinical presentation as the homozygous state of the c.14429G > A (p.R4810K) allele was highly associated with early-onset MMD (age at onset < 5 years), severe symptomatic manifestations such as cerebral infarction at diagnosis and cognitive impairment in long-term outcome [117]. There are different, partially contradictory reports on the angiographic findings in Japanese and Korean patients. Miyatake et al. reported that PCA involvement in p.R4180K homozygous patients and bilateral vasculopathy in p.R4810K homozygous and heterozygous patients was significantly more frequent than in the other genotypes in Japanese patients [118]. Nevertheless, Kim EH et al. reported that there was no difference in Suzuki stage, PCA involvement or bilateral vasculopathy between the genotypes in Korean patients [117], whereas Kim WH et al. reported that leptomeningeal collateral flow from posterior to anterior circulation was more frequent in the RNF213-negative group than in the RNF213-positive group and PCA territorial involvement was more frequently observed in RNF213-positive than in RNF213-negative Korean patients [145]. Regarding long-term cohorts, Nomura et al. followed phenotypic parameters (recurrent stroke after initial revascularization and final modified Rankin Scale) over a long-term period after direct or combined revascularization in Japanese patients. Over a median follow-up period of 100 months, there were no significant differences among the genotypes. The study confirmed that the p.R4810K variant of RNF213 influences the phenotype at onset, but there were no significant differences between the genotypes after revascularization. The frequency of recurrent stroke was low, even in the homozygous patients. Thus, direct or combined bypass surgery is suggested to be an effective treatment for all genotypes, including homozygous, heterozygous and wild type and the RNF213 genotype may not strongly influence the long-term clinical manifestations or poor prognosis in Japanese patients with MMD [125].

In comparison to Japanese and Korean patients, Wang et al. reported that the p.R4810K homozygous and heterozygous variants were also associated with early age at onset in Chinese patients, but no significant difference was found in the severity of initial symptoms between patients carrying the RNF213 p.R4810K variant and non-carriers. Unlike homozygous Japanese patients, who displayed infarction as the most common presentation, most (66.7%) of the Chinese p.R4810K homozygous carriers initially presented with TIA. This finding was contrary to the findings in Japanese and Korean patients and was not suggestive of a correlation between p.R4810K homozygous genotype and severe clinical manifestation in Chinese patients [122]. The allele frequency of p.R4810K was significantly higher in patients presenting with ischemia than in patients presenting with hemorrhage (OR = 5.4) [127]. According to multivariate cox regression analysis, the p.R4810K variant was not related to recurrent stroke or the neurological status. Therefore, similar to Japanese MMD patients, the p.R4810K variant may not be associated with long-term clinical outcome in Chinese patients. Compared to p.R4810K wild-type patients, heterozygous and homozygotes patients had a lower rate of recurrent stroke and better clinical outcome after early medical and surgical interventions [146]. Thus, surgical revascularization is suggested to be an effective treatment for p.R4810K heterozygous and homozygous Chinese patients. Angiographically, some heterozygous patients with MMD were more susceptible to PCA involvement than patients carrying wild-type alleles [122]. When comparing collateral vessels (lenticulostriate vs. thalamic vs. choroidal) in hemorrhagic hemispheres in heterozygous and wild-type p.R4810K variant patients, lenticulostriate anastomoses were found more frequently in heterozygous patients, thus lenticulostriate anastomoses might be associated with the p.R4810K variant in Chinese patients [147]. Compared to the wild-type p.R4810K variant, Chinese patients carrying the heterozygous p.R4810K variant showed better postoperative collateral formation after direct/indirect/combined bypass surgery [148].

Besides this RNF213 p.R4810K dose effect, mutations in different sites of RNF213 may have various effects on blood vessels. The RNF213 p.R4810K mutation may be the main cause of MMD intracranial artery stenosis and is more related to the MMD ischemic phenotype, whereas the rarer RNF213 p.A4399T mutation is more related to the MMD hemorrhagic phenotype [127, 149]. Apart from MMD, RNF213 variants have also been described in other cerebrovascular diseases such as intracranial aneurysms and intracranial major artery stenosis/occlusion and different mutation sites may be involved in various cerebrovascular diseases as mutations in the ATPase domain are predominantly related to intracranial aneurysms and mutations in the RING finger domain are all related to MMD (c.11990 G > A, c.12020 C > G, c.12037 G > A, and c.12055 C > T) [127, 129, 135, 149, 150]. In other diseases than MMD, RNF213 variants also lead to a more severe clinical course, e.g., R4810K carrier status was independently associated with recurrent cerebrovascular events in patients with intracranial atherosclerosis [151] and the p.R4810K variant was associated with poor clinical outcomes in patients suffering idiopathic pulmonary arterial hypertension [152]. De novo variants in RNF213 lead to early-onset and severe MMD with involvement of the abdominal aorta and renal, iliac, and femoral arteries. These variants are located in two sites: the first region is in the RING domain and the second region is a highly conserved region that is located 35 amino acids downstream from the RING domain. These de novo variants all disrupt highly conserved amino acids and thus lead to severe early-onset MMD [134]. In summary, dosage of the RNF213 p.R4810K variant appears to influence the clinical phenotype and postoperative outcome but not the long-term prognosis of MMD patients with differences between Japanese/Korean and Chinese patients, which is why it is suggested that RNF213 mutational analysis should form part of the diagnostic workup for MMD in clinical practice. The genotype–phenotype correlation of rare RNF213 variants other than p.R4810K has not been well studied in large patient cohorts in neither Asian nor Caucasian patients but different mutation sites and rare de novo variants lead to varying MMD phenotypes and different mutation sites may be involved in various cerebrovascular diseases.

### Experimental Research

After RNF213 has been described as a susceptibility gene for MMD, several groups conducted experimental research on the possible role of RNF213 in the pathophysiology of MMD. RNF213 is expressed throughout the body in humans and mice with the highest expression in immune tissues [64, 65]. RNF213 encodes the 591-kDa protein “ring finger protein 213” with two AAA + modules (ATPases associated with diverse cellular activities) and a single RING finger ubiquitin ligase domain. The RNF213 protein shows a diffuse distribution in the cytosol and is partly associated with undetermined intracellular structures. RNF213 has the ability to form toroidal oligomers and to hydrolyze ATP [116], whereas its function and link to MMD still remains elusive. It remains unclear whether p.R4810K leads to gain or loss of function of RNF213. The p.R4810K variant did not affect the transcription level, the ubiquitin ligase activity, the intracellular distribution or the stability of the protein. Knockdown of RNF213 in zebrafish caused irregular wall formation in trunk arteries and abnormal sprouting vessels in the head region [65]. Sonobe et al. generated RNF213-deficient mice (RNF213-/-) which did not develop MMD: no significant difference was observed in MRI-angiography findings, the anatomy of the circle of Willis or vascular wall thickness. However, the intimal and medial layers of the common carotid artery (proximal to bifurcation) were significantly thinner after ligation of the same in RNF213-/- mice, thus RNF213 abnormality may affect vascular wall remodelling under ischemic conditions [153]. Transient middle cerebral artery occlusion (tMCAO) in RNF213-/- mice compared to wild-type littermates did not show any cerebrovascular differences [154]. Thereafter, the same group generated RNF213-knock-in mice, resulting in a mouse featuring RNF213 p. R4828K, corresponding to p.R4859K in MMD patients. Again, RNF213-knock-in mice did not spontaneously develop MMD [155]. Morimoto et al. published results of bilateral common carotid artery stenosis surgery in RNF213-/- mice and vascular endothelial cell-specific RNF213 mutant (human p.R4810K orthologue) transgenic (EC-Tg) mice, revealing a significantly stronger reduction of CBF in the RNF213-/- mice than in wild-type mice [156]. Induced pluripotent stem cells (iPSCs) have been generated by several research groups [157–163]. Angiogenic activities of iPSC-derived vascular endothelial cells (iPSECs) from carriers and patients were lower than those of wild-type donors [157]. Angiogenesis was significantly impaired in MMD iPSECs regardless of the presence of angiogenic factors such as VEGF, whereas endothelial proliferation was not attenuated [158]. Although there is increasing evidence of reduced angiogenesis by RNF213 alterations, it remains difficult to interpret how reduced angiogenesis leads to an abnormal vascular network in MMD patients. In further cell-culture based experiments, knockdown of RNF213 in cultured endothelial cells led to the decrease of endogenous expression of cell cycle-promoting genes and less proliferative and angiogenic profiles [164]. The ratio of regulatory T cells after the administration of muramyl dipeptide (MDP)-Lys (L18) was significantly decreased in RNF213-deficient mice [165] and RNF213 was up-regulated by interferon beta [166], both suggesting a further involvement of RNF213 in the regulation of the immune system. RNF213 down-regulated expressions of MMPs in endothelial cells and thus controls the expressions of MMPs as an upstream regulator [164] and MMP-9 expression was significantly higher in RNF213-/- mice than in wild-type mice after common carotid artery ligation [167]. Through these effects on MMPs, RNF213 might play a potential role in the pathogenesis of the pathological hallmarks 1–3. Experimental research on RNF213-dependent effects inconsistently shows vascular changes without establishment of a representative MMD animal model. Cell culture experiments, including induced pluripotent stem cell derived vascular endothelial cells, are a promising approach to investigate the gap between mutations in candidate genes such as RNF213 and the development of MMD.

## Conclusion

To date, the exact pathophysiology of Moyamoya Disease has not been conclusively clarified. By evaluating the disease epidemiology with an early age of onset, familial cases and significant ethnic differences in the incidence and prevalence, a focus was put on the genetic aspects early on. The disease is characterized by certain pathological hallmarks, e.g., affection of the vessel wall and smooth muscle cells, increased angiogenic activity and altered vascular plasticity andfragility. Given these pathological hallmarks, a heterogeneous variety of genes could possibly be involved in the development of MMD. Over the last decades of genetic research, revolutionary techniques have been developed and applied, from genetic linkage studies and association studies to DNA sequencing including whole genome sequencing. These techniques have continuously yielded new results regarding the genetic basis of MMD, which in turn partially represent the pathological hallmarks. Such as association studies that revealed association to alterations in the genes of MMP, VEGF, PDGFRB, and TGFB, all substantially involved in the normal functioning of vessels. The HLA system has been studied in Asian and European MMD patients and several associations have been described. Linkage analyses verified linkage to the chromosome 17q25 and sequencing of genes encoded in this area revealed a new candidate gene, RNF213. The RNF213 founder variant p.R4810K is frequently found in East Asian (Japanese, Korean and Chinese) MMD patients and dramatically increases the risk of and susceptibility to MMD with a dose-dependent effect on clinical phenotype severity. Many non-p.R4810K variants of RNF213 and rare variants of other genes have been identified in MMD patients worldwide, but p.R4810K was found to be absent in non-Asian patients, suggesting that this variant is Asian-specific and may contribute to the high incidence of MMD in East Asia. Proof of causality and pathogenicity of these variants is still pending. RNF213 p.R4810K increases the susceptibility to MMD, however, it was also observed in 1–2% of the general East Asian population, indicating a low penetrance as many carriers are unaffected. Therefore, it is strongly suggested that in addition to p.R4810K, environmental factors play a critical role in MMD pathophysiology. Experimental research on RNF213-dependent effects inconsistently shows vascular changes without establishment of a representative MMD animal model. Cell culture experiments, including stem cell derived vascular endothelial cells, are a promising approach to investigate the gap between variants in candidate genes and the development of MMD. RNF213 is a large gene that harbours a number of variants in healthy subjects as well as in MMD patients and false attribution of pathogenicity can lead to severe consequences for patients, resulting in incorrect prognostic, therapeutic or reproductive advice. Therefore, by now RNF213 variants should be considered as susceptibility variants rather than causing variants for MMD. Future studies have to identify how different genetic pathways and environmental factors interact withRNF213 in the pathophysiology of MMD.

## Data Availability

The authors provide data transparency.
